# Characteristics of communication skills in children belonging to multispecies families

**DOI:** 10.1590/2317-1782/20232021298en

**Published:** 2023-07-21

**Authors:** Ana Paula Santa Helena, Maria Claudia Cunha

**Affiliations:** 1 Programa de Estudos Pós-graduados em Fonoaudiologia, Faculdade de Ciências Humanas e da Saúde, Pontifícia Universidade Católica de São Paulo - PUC-SP - São Paulo (SP), Brasil.

**Keywords:** Language, Language Development, Family, Dogs, Bonding, Human-Pet

## Abstract

**Purpose:**

to investigate the communicative skills of children belonging to multispecies families whose pet is a dog.

**Methods:**

this is an exploratory, descriptive, qualitative, cross-sectional study. Sample: 34 subjects of both sexes aged three aged 3 to 4 years and 5 months belonging to multispecies families. The study was conducted at the subjects’ own homes. Procedure: The data were collected through observation and filming of a 30-min interaction situation in the family routine involving the presence of the dog. Analysis of the results: The data were analyzed and content analysis categories were then established regarding the most relevant verbal and nonverbal elements, with emphasis on the child-dog-adult interlocutor communicative interactions.

**Results:**

the results showed that the dog played the role of interlocutor during the interaction scenes, with effects on the child’s communicative functions.

**Conclusion:**

the results of this study point to possible benefits to communicative skills in multispecies interactions. Further studies on this theme are suggested.

## INTRODUCTION

Humans have coexisted with dogs for thousands of years, and this initially cooperative relationship has evolved over time, establishing intense affective bonds. No longer seen as a guard animal, the dog, in addition to being a pet, is now considered a family member^(1)^.

A study conducted by the American Veterinary Medical Association^^a^^ (AVMA) shows that almost 59% of American families had a pet at the end of the 1990s, and that most of them had children^(2)^.

According to data from the Brazilian Institute of Geography and Statistics (IBGE) 44.3% of the Brazilian households have at least one dog. The dog population in Brazil was 52.2 million in 2013 - larger than that of children aged 1-14 years 44.9 million)^(3)^.

A multispecies family is a family composed of individuals who recognize and legitimize their pets as family members^(4)^. This family configuration has been the object of recent studies.

Multispecies families present many reasons for having a pet, especially companionship and affection. The role played by pets in this context varies according to the peculiarities of the family structure and the socio-emotional aspects of its members^(5)^.

Although different species of pets are present in Brazilian households, this study addressed multispecies families whose pet is a dog. In addition to their universal presence, dogs have socio-cognitive skills that allow them to interact with humans^(1)^. Moreover, although recent studies on child development and the presence of pets in families have considered different species in their methodology, dogs have been more commonly studied because of their level of interaction and potential for reciprocity compared with those of other animals^(6)^.

Including a dog in the family structure becomes effective when people recognize its importance not only from an individual perspective, but also because of its effects on the family dynamics. Thus, in modern society, pets play different roles in the multi-stage family life cycle^(7,8)^.

The current literature shows an increased scientific production on Animal-assisted Interventions (AAI) ^^b^^ whose results point to the benefits of animal participation, especially dogs, in different therapeutic environments^(9)^.

A recent study described the positive effects of the interaction between speech therapist, patient, and dog on the verbal and non-verbal communication of institutionalized elderly people^(10)^. A study on self-reported pain sensation by hospitalized children and adolescents, the researchers observed a significant decrease of this sensation after AAI^(9)^.

Dogs are used not only in the therapeutic context, but also to assist in minimizing the effects of various types of disabilities. In this case, dogs are trained to accompany individuals with visual, hearing, or motor impairments, improving their quality of life and acting as social assistance animals^(11)^.

However, this study proposes another research perspective, namely, the effects that living with a pet may have on children belonging to multispecies families.

In this regard, a recent study suggested that living with pets may contribute to the healthy development of children and adolescents^(6)^. Other studies, also recent, stated that interactions between children and animals allow them to meet the needs of physical contact typical of childhood, in addition to providing important affective experiences, such as giving and receiving love and care and coping with the phenomena of birth and death^(12)^.

The following research question was formulated for this study: Considering that interaction with dogs tends to contribute to development of children, would it specifically affect their communicative skills?

In this context, it is important to refer to research on the effects of therapist-patient-dog interaction during speech-language pathology therapy in children with oral and/or written language disorders. The hypothesis that the dog could be a therapeutic device and thus enhance these processes has been confirmed. The clinical cases reported have demonstrated that the presence of the dog provides a real motivation to participate in the therapy; favors the therapist-patient interaction; intensifies the dialogic activity, gesturing, and efficient communicative body movement; motivates reading and writing; mobilizes the patients’ affectivity; significantly decreases the symptoms observed in oral and/or written language^(13)^.

Although the proposal of this study is based on child communicative skills, its results acquire particular relevance if this process and its possible intercurrences are considered inseparably.

In fact, it is worth presenting some theoretical and methodological considerations about the process of acquiring communicative skills. This phenomenon can be approached from three perspectives: empiricist, which considers language as a result of learning; rationalist, which sees language as innate and biologically determined; dialectical, in which language is the product of an interactional process. In the empiricist tradition, learning occurs through a combination of biological maturation, mental development, and environmental stimulation. This is the oldest strand, represented by Skinner’s view of work. The rationalist perspective, on the other hand, understands language as inherent to the biological dimension of the human species, configuring Chomsky’s approach to language. According to Chomsky’s approach, the mind is the central element. For the dialectical tradition (also called interactionist), language is associated with the interaction of the child and the environment, which has Piaget, Vygotsky, and Wallon as its most important authors^(14)^.

This study considers the interactionist approach, which is based on the assumption that the subject is able to actively interact with the environment, as well as that the latter can be modified from the action of the former. According to this perspective, language is considered the first form of human socialization and, in this context, the family plays a key role^(15)^.

The literature on the interactionist approach is vast, and attests that the family plays an important role in the process of language acquisition focused on child communicative skills. Therefore, it seems pertinent to investigate the peculiarities of this process in the context of multispecies families.

According to this hypothesis, this study aimed to analyze the communicative skills that emerge in the interactions between dogs and children belonging to multispecies families.

## METHODS

**1.** This study followed the guidelines and regulatory standards for research involving human beings of the National Health Council, resolution no. 466/12, of the Ministry of Health, and was approved by the Ethics and Research Committee of the aforementioned institution (technical opinion no. 2.736.939). The participants’ parents and/or legal guardians signed an Informed Consent Form (ICF) before to study commencement.

**2. Sample:** 34 subjects belonging to multispecies families, of both sexes, aged 3 months to 4 years and 5 months.

- **Inclusion criteria:** Children interacting with the same dog(s) since birth.
- **Exclusion criteria:** Children with family complaints or previous clinical diagnosis of cognitive, motor, sensory, and/or psychological impairment.

3. Procedure:

a. Sample selection

The subjects were selected via WhatsApp or telephone contact with the legal guardians interested in participating in the study who voluntarily responded to the disclosures on social networks or were referred by them and other researchers.

In this contact, which was always with the mothers, the previously described selection criteria were verified and the data collection process was clarified. At that time, the date and time for data collection were also agreed.

b. Data collection

**Phase 1:** Application of the Questionnaire of Characterization of Multispecies Families (QCFM) (Appendix A) developed from a bibliographical survey on instruments aimed at evaluating multispecies families and content validated by three judges with practice expertise in this type of family.

**Phase 2:** Digital camera footage of a playful interaction in a natural family routine involving the dog chosen by the subjects. The most common situations included playing with balls or other toys with the dog; feeding or grooming the animal (e.g., brushing, giving medication) and expressing (verbally and non-verbally) affection to it. This was a continuous 30 min footage taken at a minimum distance of 1 m between the camera and the subjects, moving throughout the environment when needed.

4. Data analysis:

The study population was characterized by the QCFM and the data obtained were submitted to descriptive statistics.

The most significant characteristics of the acquisition process the subjects’ communicative skills were assessed by content analysis^(16)^. The videos were analyzed and content analysis categories were subsequently established in three steps: pre-analysis, material exploration, and treatment and interpretation of obtained results. The contents related to the study objective were established according to their incidence and relevance.

## RESULTS

Chart 1 presents the sample characterization according to sex and age range.

**Chart 1:** Characterisation of the study sample

**Variable**	**Category**	**N**	**%**
Sex	Male	17	50.00
	Female	17	50.00
Age group	0-11 months	6	17.65
	12-24 months	16	47.06
	2:0-3:0 years	4	11.76
	3:1-4:0 years	4	11.76
	4:1-5:0 years	4	11.76
**Total**		**34**	100.00
Age (n=34)		**Minimum - maximum (years)**	
		0.25-4.41	

The content analysis categories are now presented according to age group with the respective examples of the speech of the children and their adult interlocutors. In these examples, the subjects were identified by their first name initials, whereas the dogs are referred to by their names.

The categories were created based on the footage considering the family interaction: parents, child, and dog(s) in each age group and their relationship with the study objective.

The interactions in multispecies families with children aged 0-11 months (Table 1) showed that both the child and the adult interlocutor present communicative intention directed to the dog. The parents’ presence, stimulating and mediating the child’s contact with the dog, stands out. It was also observed that the adult interlocutors’ speeches try to interpret the dogs’ behavior to their children.

**Table 1:** Content analysis categories regarding the 0-11 month age group

**Categories**	**Examples**
Child-to-dog communication	On the floor, positioned in front of the dog (lying down), E. stretches and swings her arms crying. The mother asks: “Are you talking to Bubu?” She continues crying and looking at the dog, when the mother asks: “Do you want to get close to him? Do you? Let me see if that’s what you want.”
Interlocutor mediates child-to-dog communication	In the room, the parents are present, the baby’s sitting on the floor together with the two family dogs. The mother offers the child a biscuit. She starts to eat it and babbles: “nhanhanha”. When, then, one of the two dogs approaches and, slyly, takes the biscuit that was in one of her hands. The mother promptly intervenes, saying: “It’s over A., you lose! You’ve lost your biscuit A.”.
Interlocutor stimulates contact between child and dog	While holding her baby to burp, the mother says: “Are you well-fed, darling? Let’s sit over there on the sofa and see if Cruzer comes with us,” referring to the dog.
Interlocutor interprets the dog’s behavior for the child	The mother, interacting with the children, throws a toy (called Genoveva) for the dog to fetch it and bring it back. The dog does not respond to the play and the mother, establishing eye contact with the child, says, “Tita doesn’t want to play.”, touching her baby afterwards.
Body contact between child and dog	Sitting on the floor with the mother and older brother, the baby spontaneously crawls towards the dog, which is lying down. Balancing himself, the baby touches one of its paws (front). The dog moves and the baby reaches out towards the other (back) paw. The dog remains lying down, E. passes his hand and pulls its fur. The baby crawls again and changes the paw to be touched, when the mother warns: “Hey, no pulling huh!”

Table 2 shows an increase in dialogic abilities compared with the previous age in multispecies interactions. The communicative intention towards the interlocutor and the dog continues present; however, at this moment, the child initiates the interaction and presents a higher level of participation in the communicative exchanges. Parental supervision is constant during the interaction between the children and the dogs, either by mediating the contact, stimulating it, or interpreting the dogs’ behavior - the presence of the adult interlocutors' speech is constant.

**Table 2:** Content analysis categories regarding the 12-24 month age group

**Categories**	**Examples**
Child-to-dog communication	The mother, her daughter, and the two family dogs are sitting outside. G. spontaneously picks up a small ball from the ground, runs towards one of the dogs, called Samanta, and says: “Oinha, oinha, oinha. A Sá.”, then she puts the ball in the dog’s mouth.
Interlocutor mediates child-to-dog communication	The father and two dogs are sitting in the doorway when the daughter approaches babbling and touches the dog’s ear. The father says: “Thor's ear”. He continues talking about the parts of the dog’s body and asking G. to show: “And Thor’s tail? And Thor’s butt? And Thor’s paw?” G. plays answering, “”The pi.” And the father continues, “And Thor’s mouth?”
Interlocutor stimulates contact between child and dog	In the living room, with the child and the dog, the mother with a plush toy in her hand says to her son: “”Throw the plush toy (cat) for Popcorn to catch. Throw it to Popcorn. Catch it Popcorn, catch it”. F. takes the plush toy and, imitating his mother, said: “Éga!”. Then his mother insists: “Catch it Popcorn. Throw it, throw it, 1, 2, 3 and here it goes”.
Interlocutor interprets the dog’s behavior for the child	A. is with her mother in her room while Max is outside at the window. A. picks up a child storybook among her toys (which she always asks for). Her mother says: “Hey daughter, this story again? That one? Can’t it be another one? What if we play something else?” Max vocalizes with a kind of howl. The mother interprets it: “Your brother doesn’t want to hear that story anymore, he’s tired of it. Just one time, okay?” The mother starts reading and Max howls, the mother laughs saying: “Not even your brother can take it anymore.”
Body contact between child and dog	M. is walking in the yard with her father, who is holding her by the hand. The dogs are walking together when the mother asks: “”Cuddle Luni, daughter. M. stretches out her arm, goes to Luni, and places both hands on its fur and then hugs it.

Multispecies family interactions of children aged 2 to 3 years (Table 3) show that the dog plays an important role as a motivator in the participation of children in communicative exchanges. The dialogical skills are present in the children’s communicative intention and, when they initiate a conversation and maintain the dialogic activity with their adult interlocutors, the dog is often a theme in their speeches. The presence and supervision of the parents are still constant, but they do not interfere as much in the interactions.

**Table 3:** Content analysis categories regarding the 2:1-3:0 year age group

**Categories**	**Examples**
Child-to-dog communication	J. is in the living room playing with her mother. She makes food dishes with her toys and serves her mother. Amora comes closer and sits under her mother’s legs. J., in turn, approaches the dog and, touching its ears, says: “”Oi Amola, oi, oi.”
Interlocutor mediates child-to-dog communication	M. is playing with a doll stroller while Bruce is lying under the table. The mother warns: “Oh, I think Bruce is sick, baby.” M. walks towards the dog and, making a come gesture with her hands, says: “Tá dodói. Come Buce, come Buce.” Thereafter, she pretends to put a bandage on it saying, “That's his dodói.” M.'s great-grandmother asks, “What’s wrong with Bruce?” M. replies, “He has injured his knee.” M. asks “bisa” for her (toy) medicine. The great-grandmother separates it and she starts a joke with her mother saying, “I’m going to take care of Buxe.” M. examines the dog’s ear pretending she’s medicating it.
Interlocutor stimulates contact between child and dog	The mother, her two daughters and the dogs, Amora and Meg, are in the living room. The mother calls her daughter, who is interested in the video game: “Come here J.! Come and talk to Amora.” The sister, sitting on the floor with the dogs, says, “Hi Amora!” J. approaches the dog saying, “Hi Amola! Amolaaa! Amola is very crazy, she is crazy, crazy, crazy!” The mother asks J., “What color is Amora, daughter?” J. replies: “Black.” Her mother continues: “What color?” J. replies again: “Brown.”
Interlocutor interprets the dog’s behavior for the child	H. is in the living room with her parents and Alecrim, which is lying on the sofa. The mother looks at H. and says: “I think he wants a hug.” H. reaches to Alecrim, gives it a hug, and lays her face on Alecrim’s snout.

Table 4 shows the permanence of dialogic abilities in multispecies family interactions. There is the presence of the child’s intentional communication, as well as motivation in the communicative exchanges, in which turn taking in the interaction with the adult interlocutor become more evident. In this age group, it is clear that the children start participating in the care of their pets (feeding or daily walking) and begin to interact with them beyond play.

**Table 4:** Content analysis categories regarding the 3:1-4:0 year age group

**Categories**	**Examples**
Child-to-dog communication	V. is in the backyard with the dogs. He finds one of the toys, picks it up, and shows it to Mike shaking it: “Here, here, here.” The dog shows no interest and V. runs away.
Interlocutor mediates child-to-dog communication	L. and her mother are walking in the woods with Lola (dog). While holding the leash, L. says: “Mom, have you seen Lola “chelando” the garbage? The mother tranquilizes her daughter: “I’ve seen it.” They continue the walk when Lola leaves the sidewalk and comes to the grass. L. pulls the dog back to the sidewalk saying to her mother: “She needs to come to the sidewalk, otherwise she will get her paws dirty”. The mother again tranquilizes her daughter, saying: “We’ll clean them up later.” Then she switched positions with her daughter while walking on the sidewalk so that Lola can walk on the grass, saying: “Lola wants to venture out onto the grass because it has more smells than the sidewalk.”
Interlocutor stimulates contact between child and dog	B. is in the living room with his brothers, his mother, and Ozzy (dog). His mother asks him, “Call the dog, call Ozzy.” B., who was holding a kind of toy trumpet in his mouth, repeats: “”Ozzy, Ozzy, Ozzy, Ozzy.” His mother laughs.
Interlocutor interprets the dog’s behavior for the child	L. is with her mother and her dog (Lola) taking their daily walk in the woods. L. is walking the dog when it starts to smell the ground. The mother explains: “Lola wants to poop, wait.” L. bends down and says, “No poop?” Her mother carries on saying, “Oh, it is tough for her, Lola has not eaten any fruit, right? Did you share your fruit with Lola?” L. answers: “I did.” Her mother explains: “Her bowels are stuck, darling.”
The child performs some care toward the dog	During a walk in the streets with Lola, L. says to her mother: “I am holding the dog’s leash. She will not get far from me”. Her mother asks: “No? And why not?” L. answers: “”Because otherwise someone will take her away.” Her mother continues: “And can someone take her away?” L. adds: “Otherwise, the person will take her away and there will be no more dogs in our house.” The mother asks L.: “And would you miss Lola?” L: “Yes.” The mother concludes: “Then we have to take care of her, right? “

In the 4-5 year age group (Table 5), a greater independence of the children in the interaction with the dogs is observed. As in the previous age groups, there is permanence of the child’s communicative skills and functions. Reduced intermediation of the adult interlocutor in the child-dog relationship and the child’s ability to interpret the animal’s behavior are observed.

**Table 5:** Content analysis categories regarding the 4:1-5:0 year age group

**Categories**	**Examples**
Child-to-dog communication	L., who was in the yard telling the story of Little Red Riding Hood, runs into the house carrying a children’s book. She sits down on the floor, next to Pity. The mother says: “And Pity was waiting. Pity likes a story.” L. opens the book and says: “Once upon a time there was a Beauty.” She hugs the dog and then shows it the book so that Pity can look at the pictures. Pity moves away from L. and Nina gets closer. The mother says, “Tell Nina the story.” L. continues, with the book open: “Look over there, Nina.”
Interlocutor stimulates contact between child and dog	With her mother and the two dogs outside the house, G. comments: “They like Danone, don’t they mom?”. Her mother replies: “Do you want to give it to them? It’s ok.” G. replies: “I’ll give it to Magoo and you give it to Astro.” Mother and daughter go to the fridge, get the two yogurts. G. runs and offers it to the dog, saying: “Eat it Magoo.” She continues, “He gets all dirty! Here, take it!”
The child performs some care toward the dog	G.'s mother opens a cabinet and gets some saline solution and gauze. G. runs behind her, takes the saline solution saying: “I pour this water and mum wipes it with a cloth.” They go to the dogs and, while the mother holds one of them, G. passes the saline solution. The mother comments: “He has a lot of sleep, right?” G. says, “Now Astro, right? Wow, Astro has a lot of sleep.”
Child imitates dog’s behavior	The mother asks B. to stop playing video game and go to the backyard to play with Tita. He goes to the backyard with her, bends down, looks at her and says, “Woof, woof.” He goes back to his mum and says, “I’ve already tried.”

## DISCUSSION

The results showed both aspects of the subjects’ spontaneous linguistic performance and communicative behaviors related to human-dog interactions.

The communicative behaviors showed that the dog plays the role of interlocutor in multispecies family interactions. Moreover, it was observed that the dog’s characteristics, history, and routine management are a recurrent themes in the dialogues established between family members and child^(17,18,19)^.

These data agree with the results of a study that found that the dog can facilitate some powerful dog-family interactions and reported that talking “for the dog” is a strategy to sustain the activity^(20)^.

Still regarding communicative terms, the relationship between children and animals can play an important role, named in the literature as zooanthropological pedagogy. This concept refers to the motivation for learning and to the cognitive, affective, corporal and communicative performances^(21)^. The results of this study underlined the communicative performance(s) between child-dog-interlocutor, which can favor their language development.

Another relevant result refers to the interference in the child-dog contact and the understanding of the dog’s behavior by the adult interlocutor. The results of this study underline the communicative functioning between child-dog-interlocutor that can favor the language functioning of children and their emergence as speakers. Other relevant information refers to the interference in the dog’s behavior by the adult interlocutor. This result corroborates the findings of a study that considered the supervision of these interactions of utmost importance, as well as the adequate interpretation of the dog’s communication during child-dog contact^(22)^.

From these considerations, the role of the dog as an effective resource in mediating family interactions is highlighted. This role is also observed in promoting communicative behaviors intrinsic in dialogic activities through which it was possible to observe aspects of the process of acquisition/development of communicative skills of children belonging to multispecies families. Thus, it is important to point out some peculiarities of this process in the different age groups studied.

It is known that babies first communicate with the world through crying. However, crying cannot be considered just a reflex action. It is an important communication resource for babies since it may trigger a caregiver’s response^(23)^.

Interactions with the dog of children aged 0-24 months showed that crying was present and that the children exhibited this behavior when they were excited, as a request to get closer to it. For instance, when her daughter cried and looked at the dog, E.'s mother asked: “Do you want to get close to him? Do you? Let me see if that’s what you want”.

At 3-4 months of age, babies start to babble sequences of sounds that gradually intensify until they are about 10 months old, often accompanied by gestures^(24)^. Another example: A. babbled “nha...nhanhanha” while stretching her arms towards the dog, trying to touch it. It is noteworthy that studies on acquisition of communicative skills indicate that the adult interprets gestures first and then the vocalizations^(24)^, as occurred in the case of E., when the mother, faced with the daughter’s gesture and vocalization, said: “Do you want to get close to it? Do you? Let me see if that’s it”.

Between 12 and 24 months of age, there is a gradual evolution from babbling to idiosyncratic words, and onomatopoeia production is remarkable^(24)^. The behaviors of G. (“oinha, oinha, oinha”, referring to the little ball thrown towards the dog) and F. (“éga”, referring to the little ball caught by the dog) are highlighted.

In this age group, it is worth emphasizing the child’s ability to understand routine and situational orders with two actions^(24,25)^. For instance, when M. caressed the dog upon her mother’s request.

Between two and three years old, the child is already able to generate proto-narratives^(24,25)^, as evidenced in M., when she says that the dog is sick: “He’s sick”.

Between three and four years old, the use of verb tenses begins - present, past, and future compound^(25)^, as observed in L’s speech during a walk with the dog: “She needs to come to the sidewalk, otherwise she will get her paws dirty”; “She wants to venture out onto the grass because it has more smells than the sidewalk”.

Moreover, this period is also marked by the use of articles^(25)^, as in L inquiring the mother: “Mom, have you seen Lola “sniffing” the garbage?”.

Between four and five years of age, the child’s lexicon includes from 1500 to 3000 words^(25)^. The subjects’ speeches showed sentences with a larger number of words. Thus, L. and G. expand their narratives on themes related to dogs.

The results of this study corroborate the findings of recent studies that suggest that living with pets can contribute to the overall development of children and adolescents^(6)^.

Specifically in the field of speech therapy, studies have pointed out that human-animal interactions promote and facilitate communicative behaviors in children, adults, and older people^(9,10,13)^.

As for the effects of human-animal interactions on the acquisition/development process of child communicative skills, scientific evidence needs to be further researched because of the scarcity of studies on this theme.

## CONCLUSION

The results of this study show that, in multispecies family, children and dogs interact and that dogs often play the role of communicative partners. They also show that the involvement of dogs in communicative exchanges increasingly evolves as children’s communicative skills develop, with less need for adult communicative mediation.

Therefore, the hypothesis that the dog, in a multispecies family, enhances the child’s communicative skills cannot be refuted. Thus, this study encourages open discussion on this theme and further research is of utmost importance.

## Footnotes

a The AVMA is a not-for-profit association representing veterinarians in the United States of America.

b The term Animal-assisted Intervention (AAI) is defined by the International Association of Human-Animal Interaction Organizations (IAHAIO as any intervention that incorporates animals into the fields of health and education aiming to achieve therapeutic gains in humans.

## Appendix A Questionnaire on the characterization of multispecies families

**Table:**

Name (in full) of the person responsible for filling in the questionnaire:							
How many people live in your household?							
Provide the following information about each member of your family:							
Initials	Date of birth			Kinship	Schooling		Occupation
				
				
About the child who participating in the study:							
What is the marital status of the child’s parents?							
() Single () Married or stable union () Divorced () Widowed							
Does the child attend school?							
() Yes. Since what age? _____ () No							
If the child **attends school**, please answer:							
() Part-time () Full-time							
The school belongs to:							
() Public network () Private network							
Does the child participate in any extracurricular activity?							
() Yes. Which one(s)? ________ () No							
If the child **does not attend school**, please answer:							
Does the child participate in some activity?							
() Yes. Which one(s)? ________________ () No							
Who is the responsible adult for the child’s daily care?							
___________________________________________________________________							
With respect to the responsible adult for this care, please answer:							
Age: _______ Degree of family relationship with the child: __________________________							
Level of education: ______________							
How many and which pets live in your home?							
Pets					How many?		
() Dog					()		
() Cat					()		
() Other(s)					()		
About your dog, please answer:							
Name:			Age:			Breed	
					
How long has the dog (or each of them) been in the family?							

What are the dog’s/dogs’ behavioral characteristics?							

Who is responsible for feeding the dog(s)?							
Does your dog go to the vet?							
() Yes () No							
If so, how often?							
() Half-yearly () Annually () Only when necessary							
What rooms in your house does the dog visit?							
() Access to all rooms							
() Access to some rooms							
() Access only the external area							
In which room of the house does the dog(s) sleep?							
Does your dog participate in activities with the family? If yes, which ones?							
Has the family interaction changed after the new dog’s arrival? If yes, please name the main ones you have observed.							
								Do you celebrate your dog’s birthday?							
() Always () Sometimes () Never							
Why has the family decided to have a pet?							
Has the family ever stopped doing anything because of the dog(s)?							
Do you consider your dog a member of your family?							
() Yes () No							
Has there been any change in the relationship with the dog after the arrival of the child in the family?							
