# Supplementary material for: Characteristics of communication skills in children belonging to multispecies families
Source: Codas. 2023 Jul 21;35(4):e20210298. doi: 10.1590/2317-1782/20232021298en (PMC10446751; doi:10.1590/2317-1782/20232021298en)
Supplement: Translation

# Translation: Características das habilidades comunicativas em crianças pertencentes a famílias multiespécie

**DOI:** 10.1590/2317-1782/20232021298pt

**Authors:** Ana Paula Santa Helena, Maria Claudia Cunha

**Affiliations:** 1 Programa de Estudos Pós-graduados em Fonoaudiologia, Faculdade de Ciências Humanas e da Saúde, Pontifícia Universidade Católica de São Paulo - PUC-SP - São Paulo (SP), Brasil.

**Keywords:** Linguagem, Desenvolvimento da Linguagem, Família, Cães, Vínculo Homem-Animal de Estimação

## Abstract

**Objetivo:**

investigar as habilidades comunicativas em crianças pertencentes a famílias multiespécie, nas quais o cão é o animal de estimação.

**Método:**

estudo do tipo exploratório, descritivo, transversal, de natureza qualitativa. Casuística: 34 sujeitos de ambos os sexos, na faixa etária entre três meses e quatro anos e cinco meses, pertencentes a famílias multiespécie. A pesquisa foi realizada na residência dos próprios sujeitos. Procedimento: os dados foram coletados por meio de observação e filmagem de uma situação de interação, na rotina familiar, durante 30 minutos, envolvendo a presença do cão. Análise dos resultados: os dados foram analisados por meio de categorias de análises de conteúdo quanto a elementos verbais e não verbais, privilegiando as condutas comunicativas na interação criança-cão-adulto interlocutor.

**Resultados:**

evidenciou-se que o cão desempenhou papel de interlocutor durante as cenas de interação com efeitos nas funções comunicativas da criança.

**Conclusão:**

os resultados dessa pesquisa apontam para possíveis benefícios no que se refere às habilidades comunicativas nas interações multiespécie e sugere pesquisas posteriores.

## INTRODUÇÃO

A convivência entre humanos e cães data de milhares de anos. Mas, essa relação inicialmente cooperativa ampliou-se, ao longo do tempo, para a constituição de vínculos afetivos intensos. Não mais visto como animal de trabalho ou guarda, o cão além de animal de estimação e companhia, passou a ser considerado como membro da família^(1)^.

Um estudo realizado pela *American Veterinary Medical Association* ^^a^^ (AVMA) evidencia que quase 59% das famílias americanas possuíam um animal de estimação no final da década de 90. Destas, a grande maioria tinha crianças como membros^(2)^.

Especificamente no Brasil, segundo dados do Instituto Brasileiro de Geografia e Estatística (IBGE), 44,3% dos lares brasileiros possuem, pelo menos, um cão. A população de cães em 2013 chegou a 52,2 milhões, sendo maior que a de crianças entre um e 14 anos - 44,9 milhões^(3)^.

Destaca-se que a configuração familiar considerada multiespécie é aquela composta por indivíduos que reconhecem e legitimam seus animais de estimação como membros da família^(4)^. Tal configuração tem sido objeto de estudos recentes.

As famílias multiespécie referem muitas razões para ter um animal de estimação valorizando, sobretudo, as relações de companhia e carinho. As variações do papel do animal de estimação nesse contexto, variam de acordo com as peculiaridades da estrutura familiar e aspectos sócio - emocionais de seus membros^(5)^.

Embora seja possível observar diferentes espécies de animais presentes nos lares brasileiros, nesta pesquisa optou-se por considerar as famílias multiespécie, nas quais o cão é o animal de estimação. Além de sua presença universal, os cães possuem habilidades sociocognitivas que os permitem trocas interacionais com os seres humanos^(1)^. Além disso, embora pesquisas recentes sobre o desenvolvimento infantil e a presença de animais de companhia nas famílias tenham considerado diferentes espécies em sua metodologia, os cães são os mais pesquisados, por nível de interação e potencial de reciprocidade em comparação com os outros animais^(6)^.

A inserção de um cão na família revela-se efetiva na medida em que as pessoas reconhecem a sua importância não somente na perspectiva individual, como também pelos seus efeitos na dinâmica familiar. Nessa direção, na sociedade moderna os animais de estimação exercem diferentes papéis nos múltiplos estágios do ciclo de vida familiar^(7,8)^.

Atualmente, a literatura atesta o aumento da produção científica sobre as Intervenções Assistidas por Animais (IAA)^^b^^ , cujos resultados apontam os benefícios da participação de animais, especialmente cães, em diferentes ambientes terapêuticos^(9)^.

Estudo recente descreve os efeitos positivos da interação entre fonoaudiólogo, paciente e cão na comunicação verbal e não verbal de idosos institucionalizados^(10)^. Em pesquisa sobre a sensação de dor autorreferida por crianças e adolescentes hospitalizados, pesquisadores observaram significativa diminuição dessa sensação após IAA^(9)^.

Observa-se a utilização de cães não somente no contexto terapêutico, mas também como auxiliares para minimizar efeitos de diversos tipos de deficiências. Neste caso, os cães são treinados para acompanhar indivíduos com deficiência visual, auditiva ou motora, melhorando a qualidade de vida de seus usuários e atuando como animais de ajuda social^(11)^.

Contudo, o presente estudo propõe outra perspectiva de investigação, a saber: a dos possíveis efeitos da convivência com um animal de estimação em crianças pertencentes a famílias multiespécie.

Uma pesquisa recente sugere que o convívio com animais de companhia pode contribuir para o desenvolvimento saudável de crianças e adolescentes^(6)^. Outros estudos, também recentes, afirmam que as interações entre crianças e animais proporcionam que elas satisfaçam necessidades de contato físico típicas da infância, além de proporcionarem experiências afetivas importantes como dar e receber amor, oferecer cuidados ao outro, lidar com os fenômenos de nascimento e morte^(12)^.

Aqui, formula-se a seguinte questão de pesquisa: considerando que a interação com cães tende a contribuir para o desenvolvimento infantil, tal interação afetaria especificamente as habilidades comunicativas?

Nesse contexto, cabe referir pesquisa sobre os efeitos da interação terapeuta - paciente - cão durante processos terapêuticos fonoaudiológicos de crianças com distúrbio de linguagem oral e/ou escrita. A hipótese de o cão funcionar como um dispositivo terapêutico e, desta forma, potencializar esses processos - se confirmou. Os casos clínicos relatados demonstraram que a presença do cão proporcionou maior motivação para participar da terapia; favoreceu a interação terapeuta/paciente; intensificou a atividade dialógica, a gestualidade e a movimentação corporal comunicativa eficiente; a motivação para ler e escrever, mobilizou a afetividade dos pacientes e promoveu significativa diminuição dos sintomas manifestos na linguagem oral e/ou escrita^(13)^.

No presente estudo, embora a proposta seja focar nas habilidades comunicativas das crianças, como já citado; os resultados apresentados pela autora adquirem particular relevância se considerarmos a indissociabilidade entre tal processo e suas possíveis intercorrências.

Antes de prosseguir, vale apresentar algumas considerações teórico-metodológicas sobre o processo de aquisição das habilidades comunicativas. Tal fenômeno pode ser abordado a partir de três vertentes: a empirista, que considera a linguagem como resultante da aprendizagem; a racionalista, que compreende a linguagem como inata e biologicamente determinada; e a dialética, segundo a qual a linguagem é produto de um processo interacional. Na tradição empirista a aprendizagem se dá pela combinação da maturação biológica, desenvolvimento mental e estimulação ambiental. É a vertente historicamente mais antiga, representada pela visão skinneriana. Já a tradição inatista compreende a linguagem como inerente à dimensão biológica da espécie humana, configurando a abordagem Chomskiniana, na qual a mente é elemento central. Para a tradição dialética (também denominada interacionista), a linguagem constitui-se na interação da criança com o meio. Piaget, Vygotsky e Wallon são seus principais representantes^(14)^.

No presente estudo, assume-se a abordagem interacionista, que parte do pressuposto de que o sujeito é capaz de interagir ativamente com o meio, bem como este se modifica a partir da sua ação. Nessa perspectiva, a linguagem é considerada como a primeira forma de socialização humana e, neste âmbito, a família tem papel fundamental^(15)^.

A literatura sobre a abordagem interacionista é vasta, e atesta o protagonismo do contexto familiar no processo de aquisição das habilidades comunicativas da criança. Sendo assim, nos parece pertinente, investigar as peculiaridades de tal processo no contexto de famílias multiespécie.

A partir de tal hipótese o objetivo deste estudo é analisar as habilidades comunicativas que emergem nas interações entre cães e crianças pertencentes a famílias multiespécie.

## MÉTODO

**1.** Este estudo seguiu as diretrizes e normas regulamentadoras de pesquisas envolvendo seres humanos do Conselho Nacional de Saúde, resolução nº 466/12, do Ministério da Saúde e foi aprovado pelo Comitê de Ética e Pesquisa da instituição de origem (parecer: 2.736.939). Participaram da pesquisa os sujeitos autorizados pelos pais, através do Termo de Consentimento Livre e Esclarecido (TCLE).

**2. Casuística:** 34 sujeitos pertencentes a famílias multiespécie, de ambos os sexos, na faixa etária entre três meses e quatro anos e cinco meses.

- **Critérios de inclusão:** crianças que interagem com o mesmo cão (um ou mais) desde o nascimento.
- **Critérios de exclusão:** crianças com queixas familiares ou diagnósticos clínicos prévios de alterações cognitivas, motoras, sensoriais e/ou psíquicas.

**3. Procedimento**:

a. Seleção dos sujeitos

A seleção dos sujeitos foi realizada por meio de contato via WhatsApp ou telefônico com os responsáveis interessados em participar da pesquisa, que voluntariamente responderam às divulgações feitas em redes sociais ou foram indicados pelos mesmos e por outros pesquisadores.

Nesse contato, sempre com as mães, foram verificados os critérios de seleção acima descritos e feitos os esclarecimentos sobre o processo de coleta de dados. Nesta ocasião, data e horário para realização da coleta também foram acordados.

b. Coleta de dados

**Fase 1:** aplicação do Questionário de Caracterização das Famílias Multiespécie (QCFM) (Apêndice A), elaborado a partir de levantamento bibliográfico de instrumentos destinados à avaliação de famílias multiespécie e submetido a validação de conteúdo realizada por três juízes com *expertise* no trabalho com esta configuração familiar.

**Fase 2:** filmagem, utilizando câmera digital, de uma situação de interação lúdica, nos contextos familiares rotineiros, a critério dos sujeitos e envolvendo a presença do cão. As situações mais comuns foram: brincadeiras com bolinhas ou outros brinquedos com o cão; alimentação ou manejo com o animal (ex.: escovação, oferta de medicação) e expressões (verbais e não verbais) de carinho para com o cão. A gravação teve duração de 30 minutos ininterruptos, com distância mínima entre câmera e sujeito de um metro e deslocamento em casos de mudança de cômodo da casa por parte dos sujeitos.

**4. Análise dos resultados:**

- Caracterização da população: obtidos por meio do QCFM e submetidos à análise estatística descritiva.
- As características mais significativas do processo de aquisição das habilidades comunicativas dos sujeitos foram avaliadas por meio da análise de conteúdo^(16)^. Os vídeos foram analisados e categorias de análise de conteúdos *à posteriori* foram estabelecidas. Em três etapas (pré-análise, seleção do material e categorização do material selecionado), os conteúdos relacionados ao objetivo do estudo foram estabelecidos de acordo com sua incidência e relevância.

## RESULTADOS

A amostra é apresentada no Quadro 1, contendo sexo e faixa etária dos sujeitos.

**Quadro 1:** Caracterização da amostra do grupo pesquisa

**Variável**	**Categoria**	**N**	**%**
Sexo	Masculino	17	50,00
	Feminino	17	50,00
Faixa etária	0 a 11 meses	6	17,65
	12 a 24 meses	16	47,06
	2:0 a 3:0 anos	4	11,76
	3:1 a 4:0 anos	4	11,76
	4:1 a 5:0 anos	4	11,76
**Total**		**34**	100,00
Idade (n=34)		**Mínimo - máximo (anos)**	
		0,25 - 4,41	

A seguir, serão apresentadas as categorias de análise de conteúdo, categorizadas por faixa etária, com os respectivos exemplos de discurso das crianças e seus interlocutores adultos. Nos exemplos, os sujeitos foram identificados pela inicial do primeiro nome e os cães, pelo seu próprio nome.

As categorias foram elaboradas a partir das filmagens, considerando a interação familiar: pais, criança e cão (s) em cada faixa etária, e sua relação com o objetivo da pesquisa.

A partir das interações observadas nas famílias multiespécie com crianças na faixa etária de zero a 11 meses (Tabela 1), constata-se que a intenção comunicativa direcionada ao cão é presente, tanto por parte da criança, quanto do interlocutor adulto. Destaca-se a presença dos pais, estimulando e intermediando o contato da criança com o cão. Também se evidencia, na fala dos interlocutores, a interpretação do comportamento dos cães para as crianças.

**Tabela 1:** Categorias de conteúdo da faixa etária de 0 a 11 meses

**Categorias**	**Exemplo**
Comunicação da criança dirigida ao cão	No chão, posicionada na frente do cão (deitado), E. estica e balança os braços choramingando. A mãe pergunta: “Você está falando com o Bubu? ” Continua chorando e olhando para o cão, quando a mãe questiona: “Você quer chegar perto dele? Você quer? Deixa eu ver se é isso.”
Interlocutor intermedia o contato da criança com o cão	Na sala estão presentes os pais, o bebê sentado no chão e os dois cães da família. A mãe oferece à criança um biscoito. Ela passa a comê-lo e balbuciar: “nhanhanha”. Quando, então, um dos dois cães aproxima-se e, sorrateiramente, abocanha a bolacha que estava em uma de suas mãos. A mãe prontamente intermedia, dizendo: “Já era A., perdeu! Perdeu sua bolacha A.”.
Interlocutor estimula o contato entre a criança e o cão	A mãe, fazendo seu bebê arrotar, diz: “Está bem alimentada amor? Vamos sentar ali no sofá então e ver se o Cruzer vem com a gente”, referindo-se ao cão.
Interlocutor interpreta o comportamento do cão para a criança	A mãe, em uma situação de interação com as crianças, joga um brinquedo (chamado Genoveva) para que o cão busque e traga de volta. O cão não responde à brincadeira e a mãe, estabelecendo contato visual com a criança diz ao bebê: “A Tita não quer brincar. ”, tocando seu bebê na sequência
Contato corporal da criança com o cão	Sentada no chão com a mãe e o irmão mais velho, o bebê espontaneamente engatinha na direção do cão, que está deitado. Equilibrando-se, toca em uma de suas patas (dianteira). O cão se mexe e a criança estica a mão na direção de outra pata (traseira). O cão permanece deitado, E. passa a mão, puxa o pêlo. Engatinha novamente e muda a pata a ser tocada, quando a mãe adverte: “E., sem puxar hein! ”

Na Tabela 2, observa-se nas interações multiespécie, um crescente nas habilidades dialógicas, em comparação com a idade anterior. A intenção comunicativa para com o interlocutor e o cão continuam presentes, todavia agora a criança inicia a interação e apresenta um grau de envolvimento maior nos intercâmbios comunicativos. Observa-se que a supervisão dos pais é constante durante a interação das crianças com o cão, seja intermediando o contato, estimulando que ele aconteça ou interpretando os comportamentos do cão, a presença da fala dos interlocutores é constante.

**Tabela 2:** Categorias de conteúdo da faixa etária de 12 a 24 meses

**Categorias**	**Exemplo**
Comunicação da criança dirigida ao cão	Na área externa da casa estão presentes: mãe, filha e os dois cães da família. G. espontaneamente pega uma bolinha do chão, corre na direção de um dos cães, chamado Samanta, e diz: “Oinha, oinha, oinha. A Sá.”, em seguida coloca a bolinha na boca do cão.
Interlocutor intermedia o contato da criança com o cão	O pai e dois cães estão sentados na porta da casa, quando a filha se aproxima balbuciando e toca a orelha do cão. O pai diz: “A orelha do Thor”. Continua falando as partes do corpo do cão e pedindo para que G. mostre: “E o rabo do Thor? E o bumbum do Thor? E o pé do Thor? ” G. toca respondendo: “A pi. ” E o pai continua: “E a boca do Thor? ”
Interlocutor estimula o contato entre a criança e o cão	Na sala, com a criança e o cão, a mãe com uma pelúcia na mão diz para seu filho: “Joga o gato para Pipoca pegar. Joga para pipoca. Fala: pega Pipoca, pega”. F. pega a pelúcia e imitando a mãe, fala: “Éga! ”. Em seguida a mãe insiste: “Pega Pipoca. Joga, joga, 1, 2, 3 e já”.
Interlocutor interpreta o comportamento do cão para a criança	A. está com sua mãe no quarto, enquanto Max está do lado de fora, na janela. A. pega do meio de seus brinquedos um livro de história infantil (que costuma pedir sempre). A mãe diz: “Aí filha, essa história de novo? Esse? Não pode ser outro? E se a gente brincar de outra coisa?” Max vocaliza com uma espécie de uivo. A mãe interpreta: “O irmão também não quer mais ouvir essa história, ele já está cansado. Uma vez só, tá bom?” A mãe começa a leitura e Max uiva, a mãe ri dizendo: “Nem teu irmão aguenta mais isso aqui. ”
Contato corporal da criança com o cão	M. está andando no quintal com o pai, que a segura pela mão. Os cães passeiam junto quando a mãe pede: “Faz carinho na Luni, filha. ” M. estica o braço, vai até a Luni e passa as duas mãos no pêlo e em seguida abraça.

Observa-se nas interações familiares multiespécie das crianças na faixa etária entre dois e três anos (Tabela 3), o cão como motivador no envolvimento das crianças nos intercâmbios comunicativos. As habilidades dialógicas estão presentes na intenção comunicativa e, ao iniciar uma conversação e manter a atividade dialógica com seu interlocutor, o cão é frequentemente tema no discurso de ambos. Observa-se a presença e supervisão ainda constante por parte dos pais, porém a criança tem uma independência maior durante a interação.

**Tabela 3:** Categorias de conteúdo da faixa etária de 2:1 a 3:0 anos

**Categorias**	**Exemplo**
Comunicação da criança dirigida ao cão	J. está na sala brincando com a mãe. Faz pratos de comida com os brinquedos e serve a mãe. Amora se aproxima e senta sob as pernas da mãe. J., por sua vez, se aproxima do cão e tocando as suas orelhas diz: “Oi Amola, oi, oi. ”
Interlocutor intermedia o contato da criança com o cão	M. está brincando com um carrinho de bonecas, enquanto Bruce está deitado embaixo da mesa. A mãe avisa: “Ih, eu acho que o Bruce está dodói, neném. ” M. vai em direção do cão e fazendo gesto “vem” com as mãos diz: “Tá dodói. Vem Buce, vem Buce.” Na sequência, faz de conta que cola um curativo nele, falando: “Esse é o dodói dele.” A bisavó de M. pergunta: “O que é que o Bruce tem?” M. responde: “Fez um dodói no joelho ” M. pede para “bisa” os remédios dela (de brinquedo). A bisavó separa e ela inicia uma brincadeira com a mãe dizendo: “Vô cuidá do Buxe.” M. examina o ouvido, faz de conta que medica o cão.
Interlocutor estimula o contato entre a criança e o cão	Na sala da casa estavam a mãe, as duas filhas e os cães Amora e Meg. A mãe chama pela filha, que está interessada no vídeo game: “Vem J.! Vem conversar com a Amora.” A irmã, sentada no chão com os cães diz: “Oi Amora!” J. se aproxima do cão dizendo: “Oi Amola! Amolaaa! A Amola tá muito louca, tá louca, louca, louca! ” A mãe pergunta para J.: “Que cor que é a Amora, filha?” J. responde: “Peta.” Prossegue a mãe: “Que cor?” J. responde novamente: “Marrom.”
Interlocutor interpreta o comportamento do cão para a criança	H. está na sala com os pais e com o Alecrim, que está deitado no sofá. A mãe olha para H. e diz: “Eu acho que ele quer um abraço. ” H. vai até Alecrim, dá um abraço e encosta seu rosto no focinho do Alecrim.

Na Tabela 4, observa-se nas interações familiares multiespécie a permanência das habilidades dialógicas. Há presença da comunicação intencional da criança, bem como a motivação nos intercâmbios comunicativos nos quais ficam mais visíveis as alternâncias de turnos na interação com o interlocutor. Evidencia-se, nesta faixa etária, que a criança passa a ser participativa nos cuidados com o animal de estimação - alimentação ou passeio diário e passa a interagir além da brincadeira.

**Tabela 4:** Categorias de conteúdo da faixa etária de 3:1 a 4:0 anos

**Categorias**	**Exemplo**
Comunicação da criança dirigida ao cão	V. está no quintal de casa com os cães. Encontra um dos brinquedos, pega e mostra chacoalhando para o Mike: “Aqui, aqui, aqui. ” O cão não demonstra interesse e V. sai correndo.
Interlocutor intermedia o contato da criança com o cão	L. e sua mãe estão passeando no bosque com a Lola (cão). L. é quem conduz com cão na guia, quando fala: “Mãe, você viu a Lola chelando o lixo? ” A mãe tranquiliza a filha: “Eu vi. ” Continuam o passeio, quando Lola sai da calçada e vai para a grama. L. puxa o cão para a calçada novamente, dizendo para mãe: “Ela tem que vim pra calçada né, senão suja o pé dela”. A mãe novamente tranquiliza a filha, dizendo: “Depois a gente limpa. ” Na sequência muda a filha de posição na calçada para que a Lola possa andar na grama, dizendo: “Ela quer ir na grama porque tem mais cheiro do que na calçada.”
Interlocutor estimula o contato entre a criança e o cão	B. está na sala com os irmãos, a mãe e o Ozzy. A mãe pede para ele: “Chama, chama o Ozzy. ” B. que está com uma espécie de trombeta de brinquedo na boca, repete: “Ozzy, Ozzy, Ozzy, Ozzy. “ A mãe ri.
Interlocutor interpreta o comportamento do cão para a criança	L. está com a mãe e o cão (Lola) fazendo a caminhada diária no bosque. L. conduz o cão pela guia, quando ela começa a cheirar o chão. A mãe explica: “A Lola quer fazer cocô, vamos esperar. ” L. se abaixa e diz: “Não tem! ” A mãe continua: “Ih, tá difícil, a Lola não comeu fruta, né? Você dividiu fruta com a Lola? ” L. responde: “Dividi. ” A mãe explica: “Ela tá com a barriguinha presa filha. ”
Criança realiza algum cuidado relacionado ao cão	Durante o passeio na rua com a Lola, L. diz para sua mãe: “Eu to segurando a guia dela. Não vai chegar longe de mim”. A mãe pergunta: Não? E por que não? ” L. responde: “Porque senão alguém leva ela embora. ” A mãe prossegue questionando: “E a pessoa não pode levar ela embora? ” L. complementa: “Senão a pessoa leva e não vai mais ter cachorro lá na nossa casa. ” A mãe pergunta para L.: “E você sentiria falta da Lola? ” L: Sim. A mãe conclui: “Então tem que cuidar dela né. ”

Na faixa etária entre quatro e cinco anos (Tabela 5), observa-se uma independência maior das crianças na interação com o cão. Há permanência das habilidades comunicativas e das funções comunicativas da criança, assim como nas faixas etárias anteriores. Observa-se uma menor intermediação do adulto na relação entre criança e cão, bem como a capacidade da própria criança interpretar o comportamento do animal.

**Tabela 5:** Categorias de conteúdo da faixa etária de 4:1 a 5:0 anos

**Categorias**	**Exemplo**
Comunicação da criança dirigida ao cão	L., que estava no quintal contando a história do Chapeuzinho Vermelho, corre para dentro de casa e traz um livro infantil. Senta-se no chão, ao lado de Pity. A mãe diz: “E a Pity esperando. Essa Pity gosta de uma história. ” L. abre o livro e diz: “Era uma vez uma linda Bela. ” Abraça o cão e, em seguida, mostra o livro para que a Pity veja as figuras. Pity sai de perto de L. e Nina se aproxima. A mãe diz: “Conta pra Nina. ” Prossegue L. com o livro aberto: “Olha lá Nina. ”
Interlocutor estimula o contato entre a criança e o cão	Com a mãe e os dois cães na parte externa da casa, G. comenta: “Eles gostam de Danone né mãe?”. A mãe responde: “Você quer dar pra eles? Tudo bem então.” G. responde: “Eu dou para o Magoo e você para o Astro.” Mãe e filha vão até a geladeira, pegam os dois iogurtes. G. corre e oferece para o cão dizendo: “Come Magoo.” Continua: “Ele se suja tudo! Vai, toma!”
Criança realiza algum cuidado relacionado ao cão	A mãe de G. vai até o armário e pega o soro fisiológico e uma gaze. G. corre atrás, pega o soro fisiológico dizendo: “Eu jogo essa água e a mamãe limpa com um paninho. ” Vão até os cães e enquanto a mãe segura um deles, G. passa o soro fisiológico. A mãe comenta: “Ele tem muita remela né. ” G. diz: “Agora no Astro né. Nossa ele tem muita remela.
Criança imita o comportamento do cão	A mãe pede para que B. pare como o vídeo game e vá para o quintal da casa brincar com a Tita. Ele vai até o quintal com ela, se abaixa, olha para e diz: “Au-au, au-au.” Volta para mãe e diz: “Eu já tentei.”

## DISCUSSÃO

De acordo com os resultados, foi possível observar, tanto aspectos do desempenho linguístico espontâneo dos sujeitos quanto condutas comunicativas envolvidas nas interações humanos-cães.

Quanto às condutas comunicativas, que nas interações familiares multiespécie o cão desempenha papel de interlocutor durante as cenas de interação. Além disso, observou-se que as características do cão, sua história e o manejo da rotina também são tema recorrentes nos diálogos estabelecidos entre familiares e crianças^(17,18,19)^.

Estes dados se alinham com resultados de estudo onde se constata que o cão é um mediador potente das interações familiares. A pesquisa referida também aponta que falar “pelo cão” configura-se como estratégia para a sustentação da atividade, como constatou-se no presente estudo^(20)^.

Ainda em termos comunicativos, a relação entre as crianças e os animais pode exercer uma importante função, nomeada pela literatura como pedagogia zooantropológica. Tal conceito refere-se à motivação para o aprendizado e aos desempenhos cognitivo, afetivo, corporal e comunicativo^(21)^. Os resultados da presente pesquisa sublinham o(s) desempenho(s) comunicativos entre criança - cão - interlocutor favoráveis ao seu desenvolvimento linguístico.

Outro resultado relevante, refere-se à intermediação do contato criança-cão e da interpretação do comportamento do cão, por parte do interlocutor adulto. Os resultados da presente pesquisa sublinham o funcionamento comunicativo entre criança-cão-interlocutor favorável ao funcionamento de linguagem da criança e sua emergência como falante. Outro dado relevante se refere a intermediação do comportamento do cão realizada pelo interlocutor adulto. Tal resultado corrobora com pesquisa que considera de suma importância a supervisão dessas interações, bem como a adequada interpretação da comunicação canina durante o contato criança-cão^(22)^.

Feitas essas considerações, sublinha-se o papel do cão como recurso efetivo na mediação das interações familiares e, por extensão, na promoção de condutas comunicativas intrínsecas às atividades dialógicas por meio das quais foi possível observar aspectos do processo de aquisição/desenvolvimento das habilidades comunicativas das crianças pertencentes a famílias multiespécie. Sendo assim, cabe aqui apontar algumas peculiaridades sobre tal processo nas diferentes faixas etárias estudadas.

Sabemos que a primeira forma de comunicação dos bebês com o mundo se dá por meio do choro. Todavia, não se pode reduzir o choro apenas a um ato reflexo. Ele é um importante recurso de comunicação para os bebês, visto que, induz respostas por parte do cuidador^(23)^.

É possível observar que, nas crianças entre zero e 24 meses durante as interações com o cão, o choro esteve presente como protesto diante de algum comportamento do cão, como pedido para aproximar-se dele. Um exemplo: diante do choro e do olhar direcionado para o cão, a mãe de E. questiona: “Você quer chegar perto dele? Você quer? Deixa eu ver se é isso”.

Com três/quatro meses de idade, os bebês começam a balbuciar sequências de sons que se intensificam gradativamente até cerca de 10 meses, muitas vezes acompanhadas de gestualização^(24)^. Outro exemplo: A. balbucia “nha...nhanhanha” enquanto estica os braços para o cão, tentando tocá-lo. Salienta-se que estudos sobre aquisição de habilidades comunicativas indicam que o adulto interpreta primeiramente os gestos e, posteriormente, as vocalizações^(24)^, como ocorreu no caso de E., quando a mãe, diante do gesto e da vocalização da filha, diz: “Você quer chegar perto dele? Você quer? Deixa eu ver se é isso”.

Entre 12 e 24 meses, é notável a evolução gradativa do balbucio para palavras idiossincráticas e produção de onomatopeias^(24)^. Ressalta-se aqui os sujeitos: G. (“oinha, oinha, oinha”, referindo-se a bolinha jogada na direção do cão) e F. (“éga, para pega - referindo-se a à bolinha capturada pelo cão).

Também se sublinha nesta faixa etária, a capacidade da criança para compreender ordens rotineiras e situacionais com duas ações^(24,25)^. Por exemplo, quando o sujeito M. faz carinho no cão, a partir do pedido da mãe para que o faça.

Entre dois e três anos, a criança já é capaz de produzir proto-narrativas^(24,25)^, como evidencia-se em M., quando conta que o cão está doente: “Ele tá dodói.”

Entre três e quatro anos, inicia-se a utilização dos tempos verbais - presente, passado e futuro composto^(25)^, como observa-se na fala de L durante passeio com o cão: “Ela tem que vim pra calçada né, senão suja o pé dela”; “Ela quer ir na grama porque tem mais cheiro do que na calçada”.

Além disso, este período também é marcado pela utilização dos artigos^(25)^, também em L, quando indagando a mãe: “Mãe, você viu a Lola ‘chelando’ o lixo?”.

Entre quatro e cinco anos, o desenvolvimento lexical da criança gira em torno de 1500 e 3000 palavras^(25)^. Observa-se, nas falas dos sujeitos, enunciados com um número maior de palavras. L. e G., ampliam suas narrativas sobre temas relacionados aos cães.

Os resultados deste estudo corroboram pesquisas recentes, que sugerem que o convívio com animais de companhia pode contribuir para o desenvolvimento global de crianças e adolescentes^(6)^.

Especificamente no campo da fonoaudiologia, estudos apontam que as interações humanos-animais promovem e facilitam as condutas comunicativas em crianças, adultos e idosos^(9,10,13)^.

Quanto aos efeitos das interações humanos-animais no processo de aquisição/desenvolvimento das habilidades comunicativas infantis, as evidências científicas precisam ser mais pesquisadas; dada a escassez de estudos sobre o tema.

## CONCLUSÃO

Os resultados desse estudo evidenciam que, no lar da família multiespécie, criança e cão são parceiros interacionais e que, o cão desempenha papel de parceiro comunicativo em muitas das cenas onde a interação aconteceu. Evidencia-se também que, o envolvimento do cão nos intercâmbios comunicativos evolui num crescente, na medida em que as habilidades comunicativas da criança se desenvolvem, sendo cada vez menos necessária a mediação comunicativa do adulto.

Deste modo, a hipótese do cão, no lar da família multiespécie, potencializar as habilidades comunicativas da criança não pode ser refutada. Assim, este estudo abre discussão sobre o tema, sendo de extrema importância pesquisas posteriores.

## Footnotes

a A AVMA é uma associação sem fins lucrativos que representa médicos veterinários nos Estados Unidos.

b Utiliza-se o termo Intervenção Assistida por Animais (IAA) definido pela Associação Internacional das Organizações de Interação Humano (IAHAIO) - Animal) como toda intervenção que incorpora animais nos campos da saúde e educação com a finalidade de obter ganhos terapêuticos em humanos.

## Apêndice A Questionário de caracterização das famílias multiespécie

**Table:**

Nome (por extenso) do responsável pelo preenchimento do questionário:						
Quantas pessoas residem na sua casa?						
Sobre cada um dos integrantes da sua família, descreva abaixo:						
Iniciais	Data de nascimento	Grau de parentesco		Escolaridade		Profissão
				
				
Sobre a criança que participará da pesquisa responda:						
Qual o status marital dos pais da criança?						
() Solteiro () Casado ou união estável () Divorciado () Viúvo						
A criança frequenta a escola?						
() Sim. Desde que idade? _____ () Não						
Caso a criança **frequente a escola**, responda:						
() Meio período () Período integral						
A escola pertence a:						
() Rede pública () Rede particular						
Além da escola, a criança participa de alguma atividade extracurricular?						
() Sim. Qual? ________ () Não						
Caso a criança **não frequente a escola**, responda?						
A criança participa de alguma atividade?						
() Sim. Qual? ________________ () Não						
Quem é o adulto responsável pelos cuidados diários da criança?						
___________________________________________________________________						
Em relação ao adulto responsável por estes cuidados, responda:						
Idade: _______ Grau de parentesco com a criança: __________________________Escolaridade: ______________						
Quantos e quais são os animais de estimação vivem na sua casa?						
Animal de estimação				Quantos		
() Cão				()		
() Gato				()		
() Outro (s)				()		
Sobre seu cão, responda:						
Nome do cão:			Idade do cão:		Raça	
					
Há quanto tempo o cão (ou cada um deles) está na família?						

Quais são as características comportamentais do seu cão (ou de cada um deles?						

Quem é responsável pela alimentação do (s) cão (s)?						
Seu cão vai ao veterinário?						
() Sim () Não						
Se sim, com que frequência?						
() Semestralmente () Anualmente () Somente quando necessário						
Quais espaços da casa seu cão tem acesso livre?						
() Acesso a todos os cômodos da casa						
() Acesso a alguns cômodos da casa						
() Acesso apenas a área externa da casa						
Em qual dos cômodos da casa o (s) cão (s) dorme?						
Seu cão participa de atividades com a família? Se sim, quais?						
Ocorreram mudanças da interação da família após a chegada do cão? Se sim, cite as principais de você observou.						
							Você celebra o aniversário do seu cão? (Marque sua resposta com um X)						
() Sim, sempre () As vezes () Nunca						
Por que a família decidiu ter um animal de estimação?						
A família já deixou de fazer alguma coisa em função do (s) cão (s)?						
Você considera o seu cão um integrante da sua família? (Marque sua resposta com um X).						
() Sim () Não						
Houve alguma mudança na relação com o cão depois da chegada da criança na família?
